# Mechanical behaviour of hybrid welded-bonded joints of 3.7035 titanium alloy and CFRP in thin-walled tubular trusses

**DOI:** 10.1038/s41598-026-50989-y

**Published:** 2026-04-29

**Authors:** Cs. Farkas, D. Kollár, M. Kachichian, B. Kövesdi

**Affiliations:** 1 Genevation Aircraft Ltd, Sports Airport, 11 District II, 6078 Jakabszállás, Hungary; 2 Department of Structural Engineering, Faculty of Civil Engineering, Budapest University of Technology and Economics, 3 Műegyetem rkp, 1111 Budapest, Hungary

**Keywords:** Titanium Grade 2, CFRP, Hybrid, Mechanical properties, Thin-walled tubular, Engineering, Materials science

## Abstract

This study investigates mechanical behaviour and load-bearing capacity of welded, bonded, and hybrid welded-bonded joints made from thin-walled 3.7035 titanium alloy (Grade 2) and carbon fibre-reinforced polymer (CFRP) tubes under quasi-static loading at room temperature. The motivation lies in the aerospace industry, where significant weight savings can be achieved by replacing welded metal frames with bonded or hybrid configurations (by combining metals and composites). A comprehensive experimental program is carried out, including tensile tests on titanium base material, welded butt and T-joints, peel tests on bonded joints, combined peel-tensile tests on hybrid joints, and three-point bending tests on welded butt-joints. The results show that welded butt-joints exhibit ~ 30% lower strength than the base material, while T-joints exhibit ~ 20% lower strength than butt-joints due to non-pure tensile loading. Bonded joints demonstrate ~ 45% lower load-bearing capacity than welded joints, while hybrid welded-bonded joints show comparable strength to the base material with ~ 16% reduction relative to welded joints. Bending tests confirm the adequacy of the welding process, as no cracks or fractures are observed. The tested specimens exhibited excellent repeatability (CoV ≤ 0.10) in terms of initial stiffness and load-bearing capacity (i.e., ultimate strength and peel strength). Microscopic and macroscopic examinations, as well as hardness measurements, are conducted on welded Y-joints revealing grain coarsening and softening in the heat-affected zone, explaining the observed failure locations in welded specimens.

## Introduction

Titanium alloys are widely used due to their high strength and exceptional corrosion resistance, making them essential in aerospace, chemical processing, medical applications, and energy industry^1–4^. Titanium exhibits two allotropic crystal structures: the hexagonal close-packed α phase, stable at low temperatures, and the body-centred cubic β phase^5^, stable above the β-transus temperature *T*_β_ (~ 882 °C for pure titanium^3^ and is affected by the chemical composition^6^. Alloying elements are classified as α-stabilizers, β-stabilizers, or neutral, depending on their effect on the β-transus temperature, enabling the design of α, α + β, and β alloys, with further subcategories such as near-α and metastable β alloys^7^. The α alloys are valued for corrosion resistance and formability. The α + β alloys are the most widely used for their balanced properties, while β alloys (particularly metastable β) offer high strength and excellent hardenability^3^.

The α alloys, particularly the commercially pure (cp.) grades as the basis of the paper, are widely employed in the chemical and process engineering industries, where corrosion resistance and formability are prioritized over high specific strength^8^. Their mechanical properties are primarily controlled by oxygen content as an interstitial alloying element, which increases strength while reducing ductility. The four standard cp. grades (Grades 1–4) exhibit tensile strengths ranging from approximately 240 to 740 MPa^3,9–12^: Grade 1 offers the highest formability for deep-drawn components and cladding applications; Grade 2, the most widely used, provides a balance of strength and corrosion resistance; Grade 3, with higher strength, is suited for pressure vessels and weight-sensitive structures; and Grade 4, the one with highest strength, is generally used for fittings and mountings but requires elevated forming temperatures for complex shapes.

Most industrial titanium alloys exhibit good weldability using various fusion and solid-state techniques, including tungsten inert gas welding (TIG)^13–16^, metal inert gas welding (MIG)^17^, electron beam (EB)^18–24^, plasma^25^, laser^15,26,27^, resistance^28^, and friction welding^29–31^. Among titanium alloys, unalloyed titanium and α alloys are the easiest to weld, benefiting from their stable microstructure and low susceptibility to embrittlement, while weldability generally improves with higher α-phase content. A coarse-grained fusion zone (FZ) and a graded lamellar heat-affected zone (HAZ) generally form during welding, and their resulting properties are primarily influenced by the alloy composition^3^. The HAZ extends from the melting temperature (approximately 1668 °C^32^ at its upper limit to the β-transus temperature at its lower limit. The mechanical properties of conventionally welded titanium alloys, such as Ti-6Al-4 V, have been reported to deteriorate due to their strong affinity for reactive gases (including oxygen, nitrogen, and hydrogen) in the molten state^24^. Thus, successful welding requires stringent protection from contamination at temperatures above 250–400 °C, typically achieved through inert gas shielding or welding in a purge chamber^3^.

Numerous studies have investigated the mechanical performance of the heat-affected zone behaviour in welded titanium alloys produced by distinct welding processes. The mechanical response is primarily governed by microstructural transformations in the FZ and HAZ, particularly the formation of acicular α′ martensite during rapid cooling^22^. Several authors reported increased hardness in the FZ and HAZ compared to the base material due to martensitic transformation during TIG, laser and electron beam welding^16,26,27,33,34^. In the case of electron beam welding of Ti-6Al-4 V, increased weld zone hardness is frequently observed; however, marginal reductions in tensile strength and ductility have also been reported, attributed to microstructural refinement, martensite formation, and microsegregation effects^22–24^. Conversely, substantial softening has been documented solely in friction stir welded commercially pure titanium joints yet, where reductions in yield and tensile strength depend strongly on process parameters such as rotational and traverse speed^30,31^. In as-welded (TIG) commercially pure titanium butt-welded joints, slightly lower hardness was reported in the FZ in^13,15^.

The aerospace industry places strong emphasis on minimizing structural weight, with weight rationalization serving as a guiding principle in design and development. For both manned aircraft and unmanned aerial vehicles (UAVs), empty weight and payload capacity are key parameters. The UAVs, which were initially developed primarily for military applications^35^, have expanded into a wide range of civilian uses^36^ and are now an increasingly important part of modern and future transportation systems. In civil contexts, one of their primary functions lies in logistics: transporting small raw materials, pharmaceuticals, food products, and postal packages between distribution points or directly to end users. In order to minimize weight while maintaining overall stiffness and structural integrity, it is beneficial to employ frames with planar and spatial configurations as the main structural system, complemented by the use of composite elements in place of traditional metallic components in selected areas. In general, the state of the art in hybrid metal-composite joining has already been comprehensively reviewed in the literature^37,38^. However, the majority of published studies focus on flat specimens or lap joints with hybrid configurations, including bolted joints^39–43^, adhesive bonded joints^43–45^, penetrative reinforcement methods using pins and protrusions^45–50^, and other thermomechanical joining^51–53^ or interlocking techniques^53–58^. In contrast, hybrid joining solutions for tubular components, which are of particular relevance to lightweight UAV structures, remain only sparsely addressed in the available literature^59–63^, with existing studies covering only a limited range of configurations, hybrid joining techniques, loading conditions, and environmental effects. This indicates a clear research gap concerning the structural behaviour and applicability of such joints in tubular frame configurations. At the same time, ensuring a reliable bond between tubular titanium and composite elements requires advanced manufacturing techniques, making the fabrication of such frames considerably more complex than the purely welded counterparts.

This paper investigates the mechanical behaviour of hybrid welded-bonded joints, subject to quasi-static loading, between 3.7035 (Grade 2) titanium alloy and carbon fibre reinforced polymer thin-walled tubular trusses using experimental methods. It is a previously unexplored field that provides a foundation for incorporating these heterogeneous joints into the design philosophy of UAV aircrafts. First, the comprehensive experimental research program is introduced in Sect.  2, including the list of laboratory tests, applied materials and specimens and the characterization methodology. Then, the experimental results of the conducted tests are discussed in Sect.  3 related to the mechanical behaviour of the analysed components. In addition, the discussion is extended from the joint level to the structural integration and scalability of the tubular UAV space truss briefly in Sect.  4. Finally, Sect.  5 highlights the key findings and statements discussed throughout the study, while also providing a brief overview of potential directions for further investigations.

## Experimental research program

**Fig. 1:**
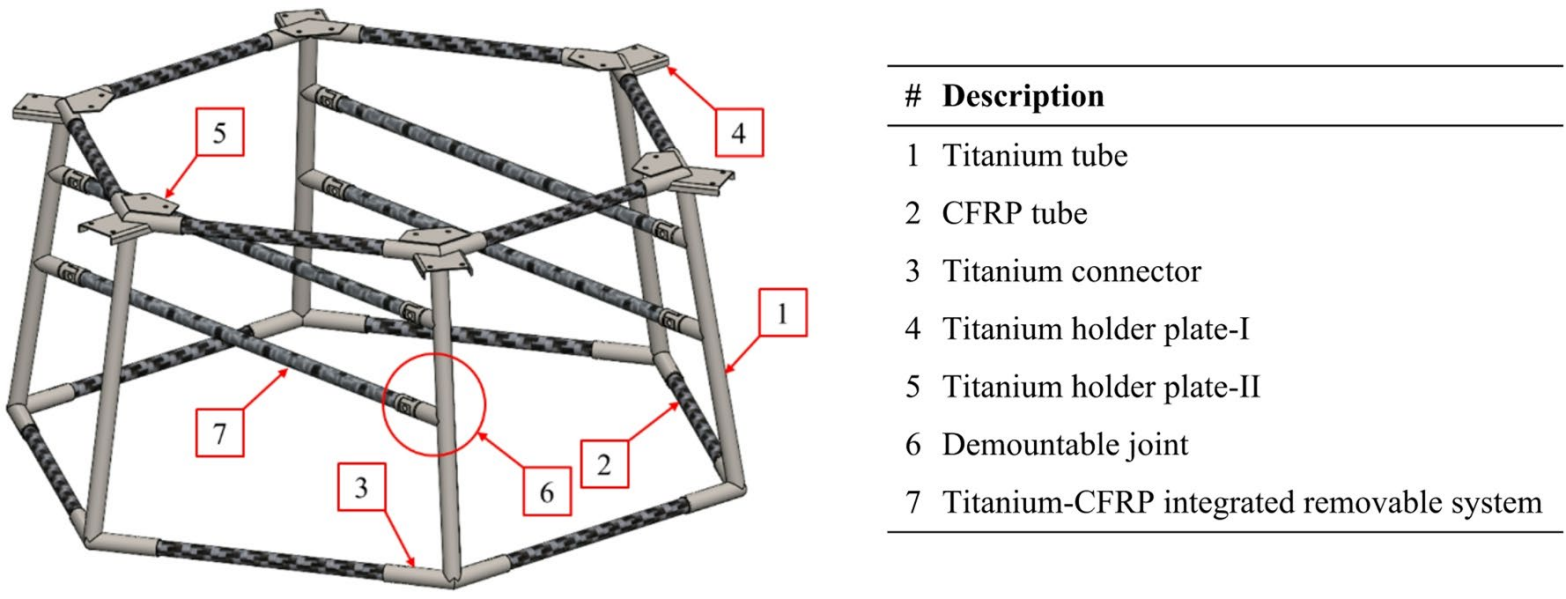
CAD model of the tubular space truss of the UAV with the analysed joints (total self-weight is 3.0 kg).

Laboratory tests are conducted to analyze specific joints of the tubular truss structure in a lightweight unmanned aerial vehicle (UAV) designed and manufactured by Genevation Aircraft Ltd. Figure 1 demonstrates the tubular space truss of the UAV and the main parts with the analysed joints. The spatial frame consists of 3.7035 (Grade 2) titanium alloy tubular hollow sections and carbon fibre reinforced polymer (CFRP) composite tubes.

The main aim of the investigations is to thoroughly assess the load-bearing capacity, material properties, and behaviour of the applied welded, bonded or hybrid welded-bonded joints subject to quasi-static loading, while investigating the influence of joining technologies. The following typical configurations are examined:

- tensile tests on base material (3.7035 titanium alloy tubular hollow sections),
- tensile tests on welded tubular butt-joints (3.7035 titanium alloy),
- tensile tests on welded tubular T-joints (3.7035 titanium alloy),
- three-point bending tests on welded tubular butt-joints (3.7035 titanium alloy),
- peel tests on bonded joints (3.7035 titanium alloy/CFRP),
- combined peel-tensile tests on hybrid bonded-welded tubular joints (3.7035 titanium alloy/CFRP/3.7035 titanium alloy),
- additionally microscopic and macroscopic examinations, and hardness measurements on welded tubular Y-joints (3.7035 titanium alloy).

### Materials and specimens

In the analysed specimens, seamless cold finished tubular sections of 3.7035 titanium alloy are used with outer diameter of *D*_Ti_ = 19.05 mm, and wall thickness of *t*_Ti_ = 1.65 mm (cross-sectional area *A*_Ti, tube_ is equal to 87.7 mm^2^). It is a single-phase (pure α-phase) titanium alloy known for its excellent corrosion resistance, good weldability, and a favourable balance of strength and ductility. Based on the inspection certificates, proof strength *R*_p0.2_ and ultimate strength *R*_m_ are 360 MPa and 502 MPa, respectively, while ultimate strain *A* is 34.5%. The guaranteed minimum proof strength and ultimate strength are *R*_p0.2,min_ = 250 MPa and *R*_m, min_ = 390 MPa, respectively. The minimum ultimate strain is 22%. Chemical composition of the titanium tubes is summarized in Table 1. Hardness of the base material is 88.28 HRB (approximately 175 HV), according to the inspection certificate.

**Table 1:** Chemical composition of titanium tube and filler material according to inspection certificates.

Material	Fe%	C%	*N*%	O%	H%
3.7035 (tube)	0.05	0.002	0.001	0.17	0.003
Requirement (DIN 17850)	≤ 0.20	≤ 0.06	≤ 0.05	≤ 0.18	≤ 0.013
AWS A5.16-90 ERTi-2 (filler material)	0.06	0.02	0.013	0.080	0.004
Requirement (AWS A5.16)	≤ 0.12	≤ 0.03	≤ 0.015	0.08 ≤ O%≤0.16	≤ 0.008

**Fig. 2:**
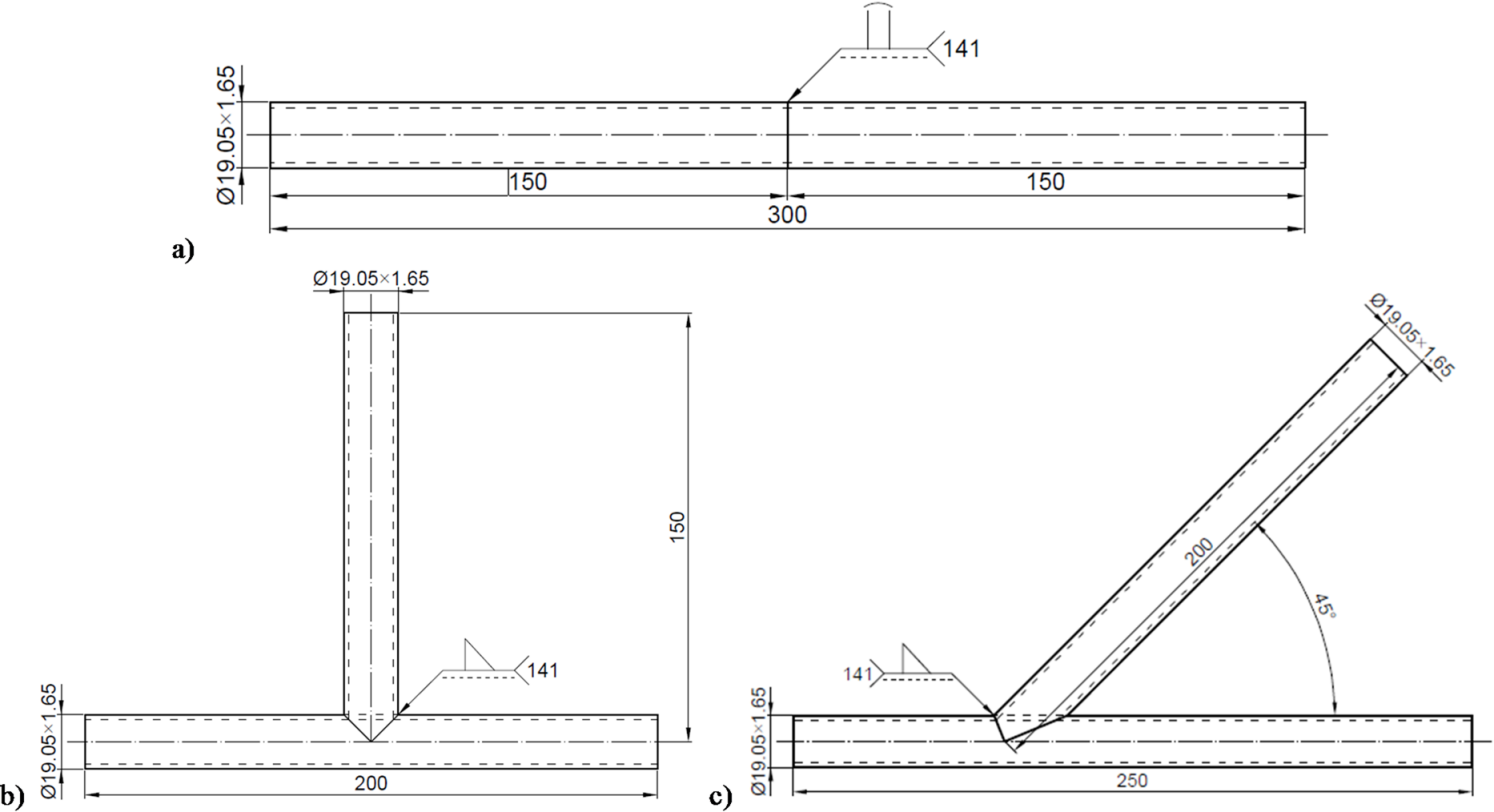
Schematic of analysed welded joints: (**a**) butt-joint, (**b**) T-joint and (**c**) Y-joint.

Laser cutting is applied for carving the tubular sections. There are three different types of welded joints: (a) butt-joint with full penetration square butt weld without edge preparation (Fig. 2a), (b) T-joint with fillet weld (Fig. 2b), and (c) Y-joint with fillet weld (Fig. 2c). Each joint is welded manually in either PA flat or PB horizontal welding position, according to EN ISO 6947, with one weld pass. The applied welding process is tungsten inert gas (TIG) welding with solid filler material (141). An ESAB Caddy TA34 welding power source is used with direct current and straight polarity (DC-). The type of the inert shielding gas during manufacturing is ISO 14,175 – I1 – Ar (Argon 5.0) with gas flow rate of 9–10 l/min. It is underscored that complete shielding gas protection is applied during welding (Argon 4.6) in a purge chamber, while backing gas (Argon 5.0) is used with gas flow rate of 4–5 l/min at the weld root within the tubes. The solid rod is AWS A5.16 ERTi-2 (a commercially pure titanium filler for Grade 2) with a minimum proof strength and ultimate strength of *R*_p0.2,min_ = 275 MPa and *R*_m, min_ = 345 MPa, respectively. The minimum ultimate strain is 20% in accordance with ASTM B348. Diameter of the solid rod is 1.6 mm for the butt-joints, and 1.0 mm for the T-joints and Y-joints. Chemical composition of the solid rod is summarized in Table 1. No preheating is applied, while ambient temperature is between 20 and 22 °C during manufacturing. For the butt welds, welding current *I* is 50 A and voltage *U* is 11.7 V, while travel speed is 5.8 cm/min resulting in net heat input *q* = η*UI*/*v* equal to 0.36 kJ/mm. Thermal efficiency η is assumed to be 0.60 for TIG welding based on EN 1011-1. For the fillet welds with throat thickness *a* of 2 mm, welding current *I* is 57 A and voltage *U* is 12 V, while travel speed is 4.5 cm/min and net heat input *q* is 0.55 kJ/mm. Welding designations, including the main dimensions, are shown for each joint in Fig. 2. The circumferential welds of the butt-joints are made in two 180° segments, positioned opposite each other on opposing sides. The T-joints and Y-joints are welded in four 90° segments, with the welding sequence carried out by alternating between opposing sides.

**Fig. 3:**
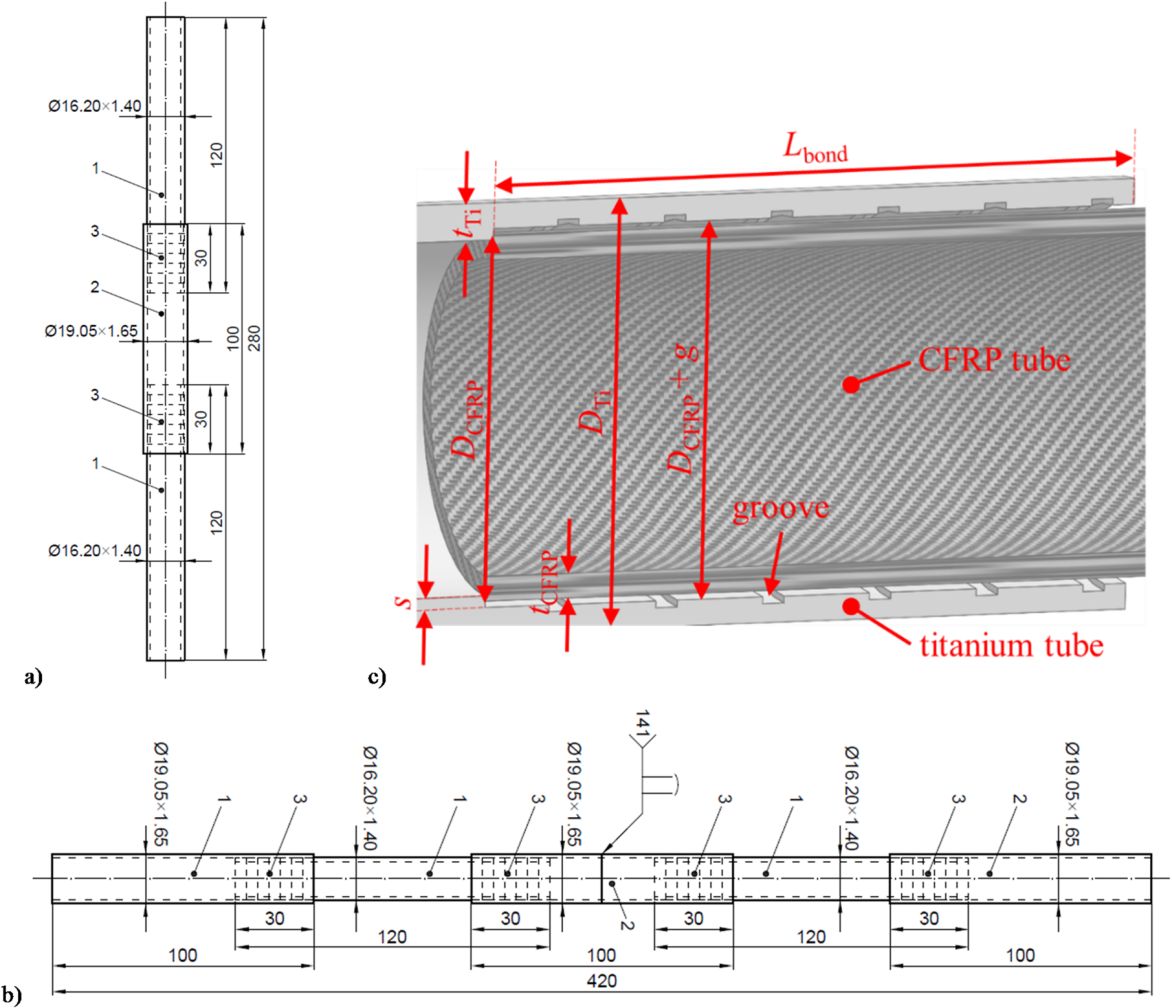
Schematic of (**a**) bonded joints, and (**b**) hybrid bonded-welded joints; (**c**) notation of geometrical parameters for bonded joints (1 – CFRP, 2 – titanium, 3 – bonded with adhesive).

In the case of bonded joints (Fig. 3a) and hybrid bonded-welded joints (Fig. 3b), the 3.7035 titanium alloy tubular sections are combined with CFRP tubes. The CFRP hollow sections are roll-wrapped carbon fibre tubes with a high-quality epoxy resin matrix, composed of multiple layers. Layer sequences and fibre orientations are given in Table 2. The 0° orientation corresponds to the longitudinal axis, representing the principal load-bearing direction. Outer diameter of the CFRP tube is *D*_CFRP_ = 16.2 mm, and wall thickness is *t*_CFRP_ = 1.4 mm. Mechanical properties of the composite and matrix are summarized in Table 3.

**Table 2:** Layers and fibre orientations in CFRP tubes.

Layer #	Description	Angle [°]	Thickness [mm]
1	250 g/m^2^ 2 × 2 twill weaves	0/90	0.15
2	300 g/m^2^ UD	0	0.30
3	150 g/m^2^ BD	± 45	0.12
4	300 g/m^2^ UD	0	0.30
5	150 g/m^2^ BD	± 45	0.12
6	250 g/m^2^ 2 × 2 twill weaves	0/90	0.15

Note: UD: unidirectional.

BD: bidiagonal (also known as ‘double bias’).

**Table 3:** Mechanical properties of the applied CFRP.

Property	Value	Direction/note
Young’s modulus *E*_0_	99 GPa	0° (longitudinal, along tube axis)
Young’s modulus *E*_90_	18 GPa	90° (transverse)
Shear modulus *G*_12_	5.8 GPa	In-plane
Poisson’s ratio ν	0.13	–
Tensile strength σ_tens,0_	860 MPa	0° (longitudinal, along tube axis)
Tensile strength σ_tens,90_	160 MPa	90° (transverse)
Compressive strength σ_comp,0_	820 MPa	0° (longitudinal, along tube axis)
Compressive strength σ_comp,90_	256 MPa	90° (transverse)
Interlaminar shear strength τ_±45_	147 MPa	± 45°

Araldite 420 A/B, a special two-component aerospace adhesive with extremely high shear and peel strength, is used between the CFRP and titanium tubes. Although the paste adhesive can cure at room temperature, it is cured at 70 °C for 6 h to enhance mechanical properties in line with the technical datasheet of the product. The temperature is gradually raised from room temperature to 70 °C in 10 °C increments, with 30 min of ramping and an additional 30 min of holding at each intermediate step (30, 40, 50, and 60 °C). According to its technical datasheet, a shear strength of 11 MPa can be guaranteed with this approach. The titanium tubes are fabricated with grooved ends (in the transverse direction) to enhance bonding performance and ensure improved mechanical interlocking at the joint interface (Fig. 3c). Bonding length *L*_bond_ between the carbon tube and the titanium tube is 30 mm. The inner surface of the titanium tube is machined with a shoulder size *s* of 0.5 mm, while a bonding gap *g* of 0.6 mm is applied.

### Characterization methodology

Due to the small tube diameter and thin wall thickness, standardized toughness tests, such as Charpy or crack tip opening displacement (CTOD), are not feasible, as valid specimens cannot be extracted from the welded joints. Therefore, weld performance and damage tolerance are assessed by joint-level quasi-static mechanical testing (tension/bending) at room temperature, supplemented by hardness measurements and metallographic examination of the weld metal and heat-affected zone. The following subsections describe the corresponding test setups, boundary conditions, loading procedures, and measurement methods for the base material as well as welded, bonded, and hybrid bonded-welded joints. Environmental effects, such as low temperature, are not considered in the current experimental program.

#### Tensile tests

**Fig. 4:**
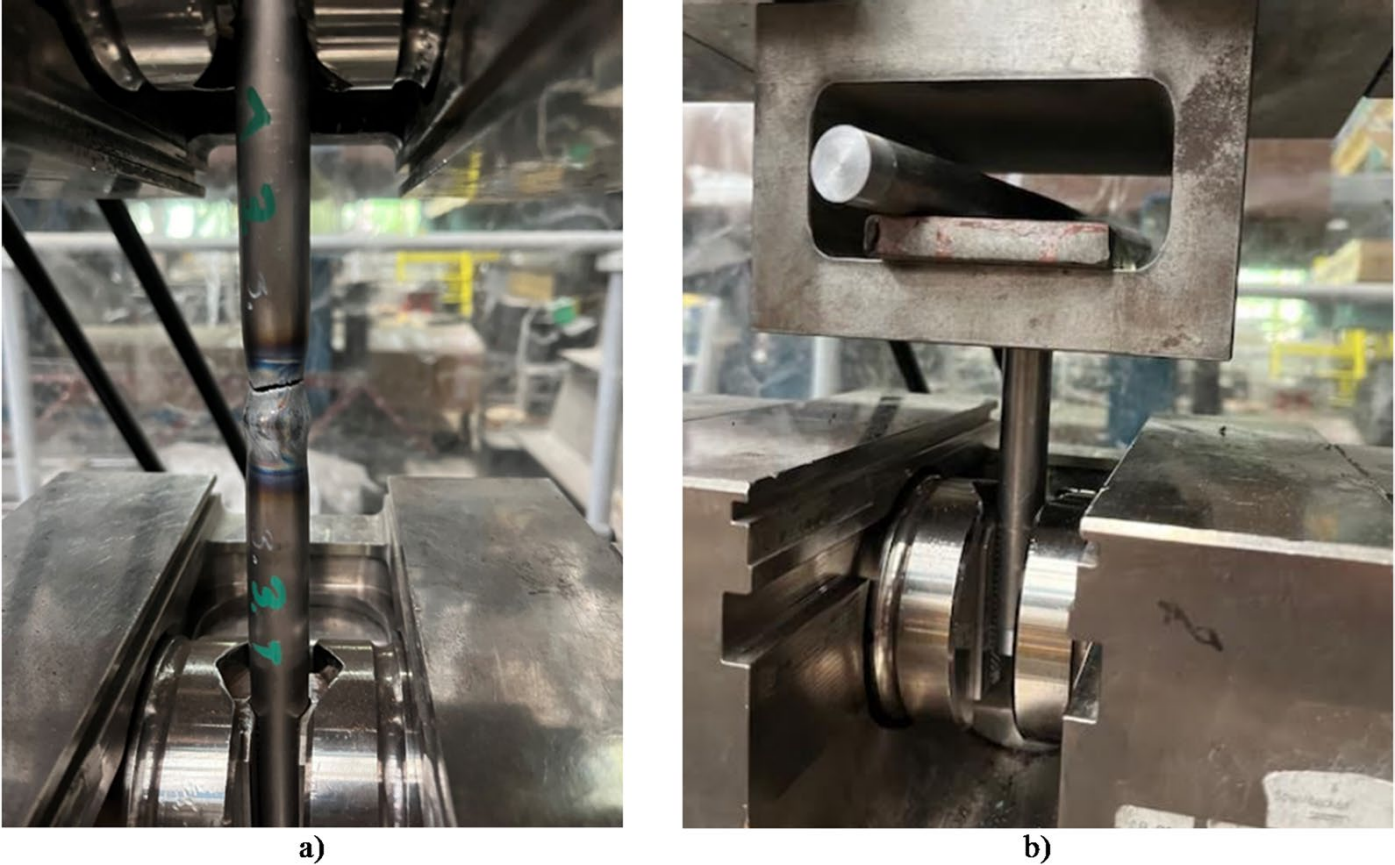
Set-up for tensile testing of (**a**) welded tubular butt-joints and (**b**) T-joints.

Tensile tests are carried out using a ZwickRoell Z400 testing machine, with a measurement capacity of 400 kN, to assess the mechanical behaviour of the titanium base material, the welded tubular butt-joints and welded T-joints. For the straight tubular specimens (base material and butt-joints), the gauge length is 200 mm within a total specimen length of 300 mm, corresponding to a gripping length of 2 × 50 mm. Hydraulic wedge grips are used for specimen clamping (Fig. 4a). For the T-joint specimens, an auxiliary plate with a drilled hole is attached at the top to ensure proper fixturing (Fig. 4b). This arrangement enabled secure load introduction without directly gripping the horizontal tube, thereby preventing local crushing and avoiding the application of clamping forces on the weld. The vertical tube at the lower end is clamped over a gripping length of 50 mm by means of hydraulic wedge grips (gauge length is approximately 100 mm). Steel plugs are inserted at the gripping areas to prevent local crushing in all the cases. Crosshead displacement is measured, which includes frame and grip compliance. Load is measured using the integrated calibrated load cell, with accuracy class 0.5 in line with EN ISO 7500-1 (relative reversibility error is ± 0.14%, relative repeatability error is 0.13% and relative measurement accuracy is 0.17% based on the calibration certificate). The crosshead speed is set according to EN ISO 6892-1 by controlling the strain rate (method A). The tests are performed at a temperature of 26 °C.

**Fig. 5:**
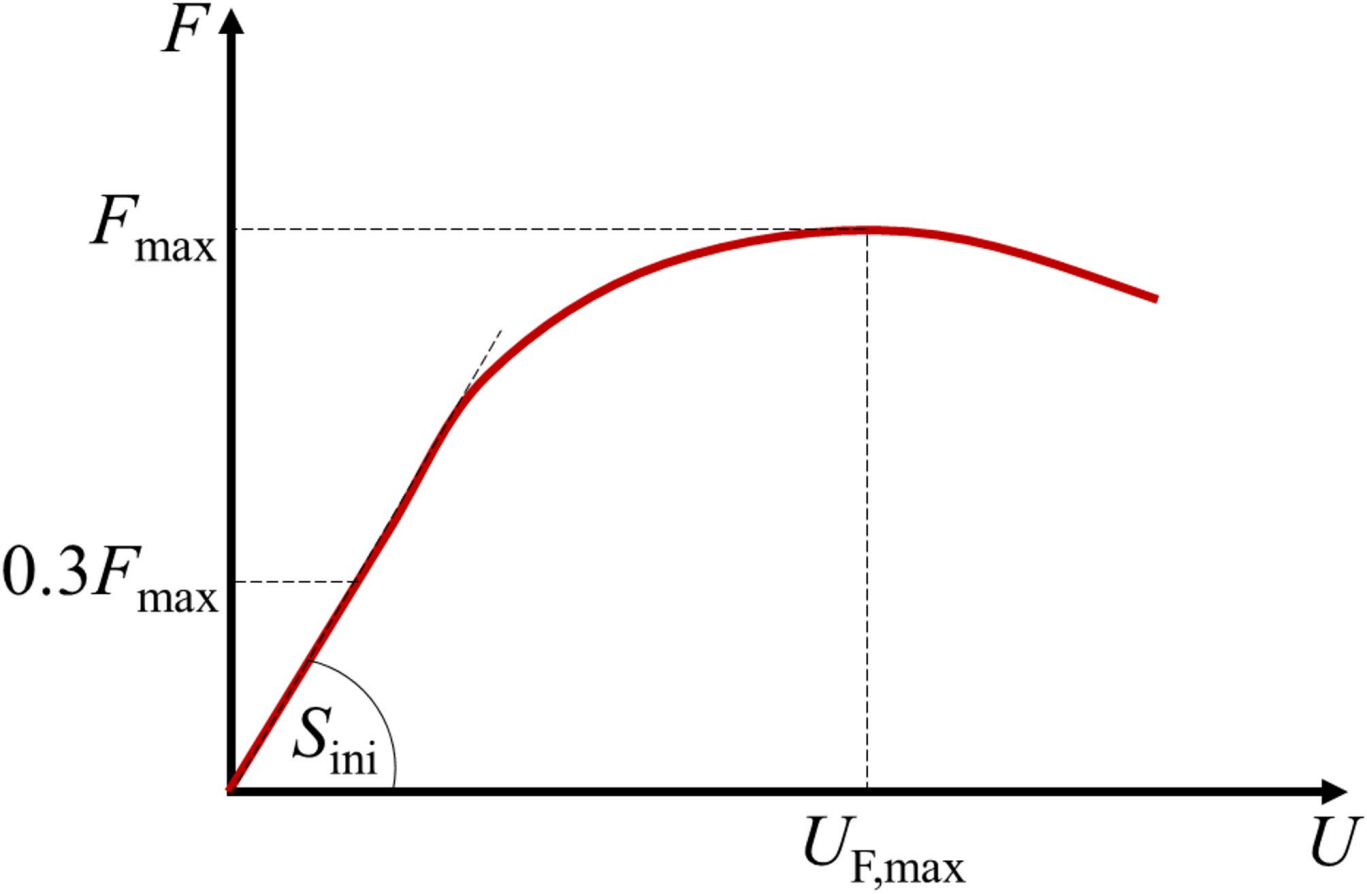
Schematic representation of the derived parameters from load-displacement (F-U) curves.

Load-bearing capacity *F*_max_ and ultimate strength *R*_m_ = *F*_max_/*A*_Ti, tube_ of the base material are determined in addition to crosshead displacement *U*_F, max_ at *F*_max_ to differentiate between the various joining technologies (Fig. 5). It needs to be underscored that CFRP behaves quite rigidly and exhibits inherently low ductility, with first fibre failure typically marking its load and deformation capacity; therefore, these parameters (*F*_max_ and *U*_F, max_) serve as the key indicators for distinguishing the different joints in the actual hybrid application. In lightweight aerospace frame design, initial stiffness is a governing parameter influencing structural performance and stability. Therefore, the initial elastic stiffness (*S*_ini_) of the different joint configurations is quantified by determining the slope of the load-displacement curves within the linear elastic region. Therefore, least-squares linear regression is applied over the initial range defined as 0–30% of the load-bearing capacity (0.3*F*_max_).

#### Peel tests and combined peel-tensile tests

In structural design, preferably in the aerospace industry, a significant weight reduction can be achieved by using bonded and hybrid (bonded-welded) joints between composite and metal parts instead of solely applying metal components and welding. Therefore, peel tests are conducted using the universal ZwickRoell Z400 testing machine on bonded joints, combining titanium tubes and carbon fibre tubes, in order to determine the peel strength τ_peel_. It is a fundamental measure for the strength design of these connections. The resistance of the adhesive bond against peeling can be calculated using τ_peel_ = *F*_max_/(*L*_bond_·*D*_bond_·П), where *F*_max_ is the maximum load, *L*_bond_ and *D*_bond_ are the bonded length and diameter, respectively. The bonded length is 30 mm, while bonded diameter is equal to *D*_CFRP_ + g = 16.2 mm + 0.6 mm = 16.8 mm in the analysed cases. The gauge length is 180 mm within a total specimen length of 280 mm (with gripping length of 2 × 50 mm). In addition, hybrid joints are created by integrating specially fitted, press-bonded metallic inserts (Fig. 3c) into high-strength carbon fibre tubes, which is crucial during manufacturing of the space frame of the UAV. These inserts are subsequently welded to the adjoining elements at their designated locations, ensuring precise alignment. To maintain metallurgical compatibility during welding, the material grade of the insert embedded within the fibre-reinforced composite tube corresponds to that of the adjacent metallic components. To evaluate the performance of this configuration, representative of actual structural application in the space frame, combined peel-tensile tests are conducted on hybrid bonded-welded tubular joints. In this case, the specimens have a total length of 420 mm, corresponding to a gauge length of 320 mm. Steel plugs are inserted at the ends of all the CFRP tubes in the gripping regions to prevent local crushing. The loading protocol, boundary conditions, and evaluation methodology are identical for the bonded and hybrid specimens. The crosshead displacement is controlled to maintain a strain rate of 0.002 s^− 1^. Specimen edges are clamped using hydraulic wedge grips.

#### Three-point bending tests

**Fig. 6:**
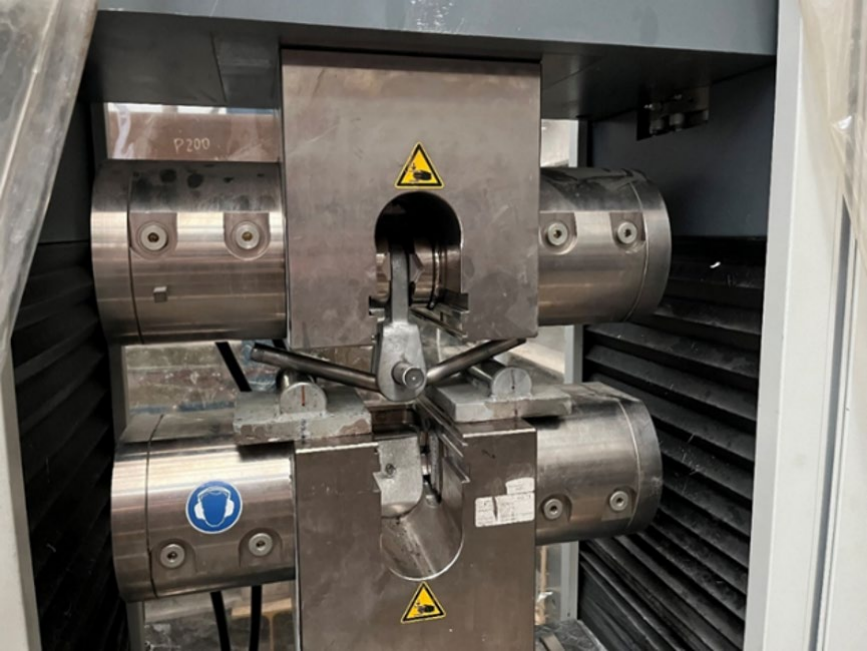
Set-up for three-point bending tests.

Three-point bending tests are carried out using the same ZwickRoell Z400 testing machine (Fig. 6). Welded tubular butt-joint specimens, with a total length of 300 mm, are tested to verify the adequacy of the applied welding procedure. To avoid local damage near the supports, 50 mm long steel plugs are inserted into both ends of the specimens. The absence of fractures or crack initiation during testing is considered indicative of satisfactory welding quality and sufficient plastic deformation capacity. Spacing *L* between the semi-cylindrical supports is 250 mm. Crosshead displacement and load are measured, while the crosshead speed is set in accordance with EN ISO 8491 and EN ISO 7438 by controlling the displacement rate to 1 mm/s.

#### Macroscopic and microscopic examinations

**Fig. 7:**
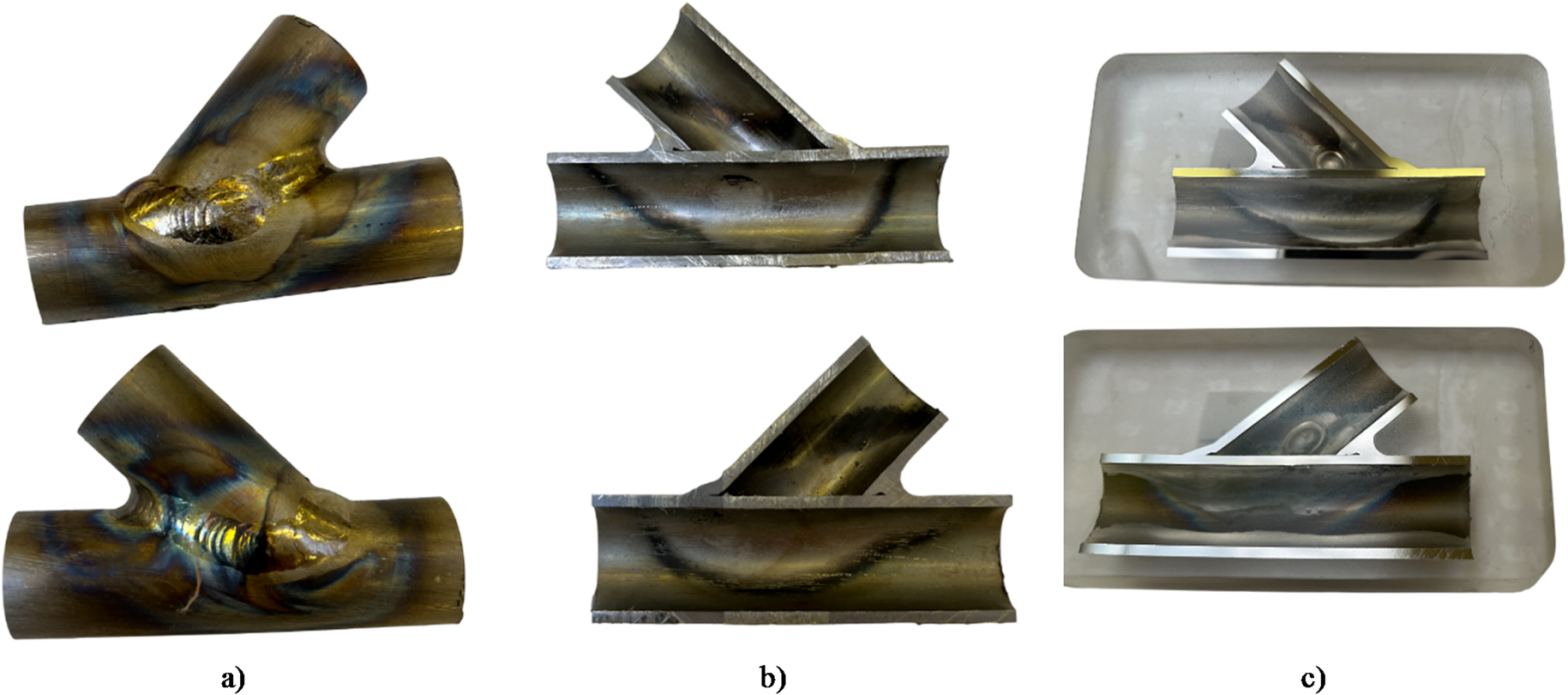
(**a**) Local weld zone after sectioning, (**b**) after longitudinal cutting, and (**c**) embedded samples in acrylic resin.

Macroscopic and microscopic examinations, including hardness measurements, are also carried out on two welded tubular Y-joints. Stereomicroscopic observations are performed with an Olympus SZX16 stereomicroscope, while optical microscopic examinations are carried out with an Olympus PMG-3 metallographic microscope. Microhardness measurements are conducted using a Buehler IndentaMet 1100 microhardness tester (HV_0.5_ is determined with *m* = 500 g). Prior to the analysis, welded joints are sectioned with a precision cutting machine, as illustrated in Fig. 7a,b, which also shows the weld crown, root, and welding-induced coloration. The heat-affected zone (HAZ) is clearly distinguishable in the sectioned samples. For metallographic preparation, the cut surfaces are degreased and embedded in Struers ClaroCit acrylic resin (Fig. 7c).

The samples are ground and polished using a Tegramin-30 machine. Initial grinding is performed with P320 SiC foil under water flow until a flat surface is obtained, followed by grinding with an MD-Mezzo disc for 2 min, also under water (this disc is specifically designed for titanium). Fine grinding is carried out on an MD-Largo disc with DiaPro All/Lar 9 μm suspension. Polishing is performed on an MD-Nap cloth with DiaPro Nap R 1 μm suspension for 4 min. To achieve a scratch-free finish, final polishing is conducted on an MD-Chem cloth using OP-S Nondry 0.25 μm suspension for 10 min. Etching is performed with Kroll’s reagent (2 ml HNO₃, 1 ml HF, 100 ml distilled water) to reveal the grain structure and to differentiate the base material (BM), heat-affected zone (HAZ), and weld metal (WM).

## Results and discussion

### Tensile tests

**Fig. 8:**
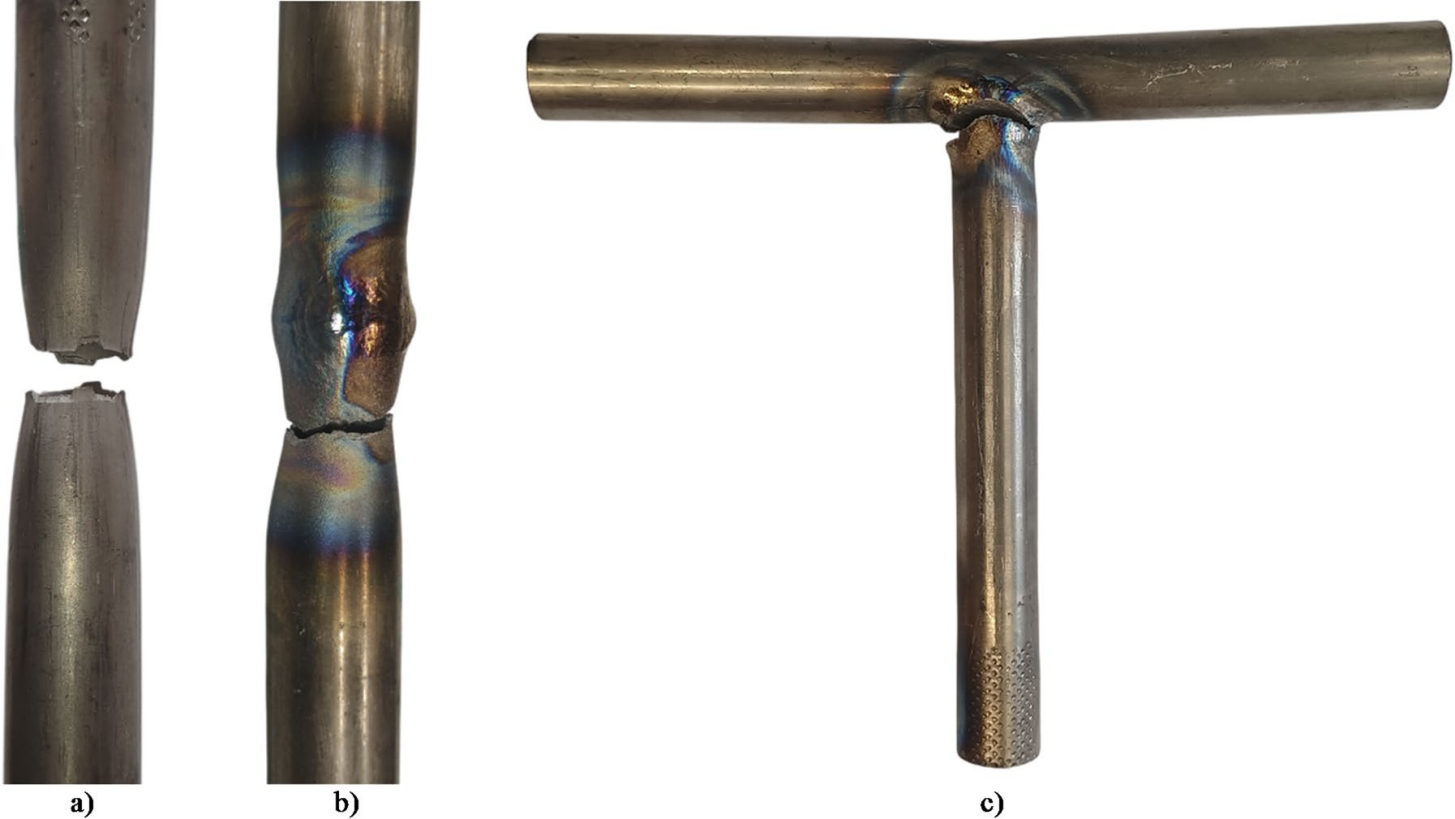
Typical failure modes of (**a**) base material, (**b**) welded tubular butt-joint, and (**c**) T-joint.

**Fig. 9:**
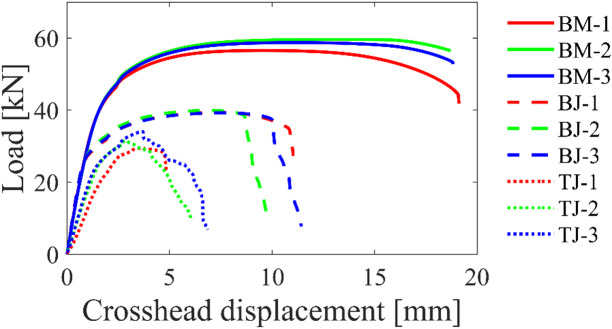
Measured load-displacement curves during tensile tests.

Firstly, three tubular sections fabricated from 3.7035 titanium alloy, free of any welded or bonded joints, are examined to characterize the properties of the base material (BM-1 to BM-3). Ultimate strength varies between 645 and 680 MPa, with necking and ductile failure (Fig. 8a), which is significantly higher than the minimum guaranteed value (390 MPa). Secondly, the welded tubular butt-joints (BJ-1 to BJ-3) are tested. Ultimate strength ranges between 448 and 455 MPa, with a mean value of 451 MPa, which shows approximately 30% reduction compared to the strength of the base material. Nevertheless, it is still higher than the minimum strength which needs to be guaranteed according to the DIN 17,861 standard. The predominantly ductile failure, characterized by progressive load increase and moderate elongation capacity compared to the base material prior to failure, consistently occurs outside the weld indicating heat-affected zone-governed failure (Fig. 8b). Owing to the relatively low thermal conductivity of titanium, the heated area cools more slowly, which can lead to grain coarsening and softening (discussed further in Sect.  3.4). Finally, welded T-joints (TJ-1 to TJ-3) are examined showing ductile tearing near the weld toe (Fig. 8c). The mean ultimate strength is 362 MPa, which is approximately 20% lower than that of the butt-joints. This reduction is attributed to the non-axial loading condition of the T-joint specimens, where the fixturing introduces a slight bending component (the inherent bending deformation of the horizontal tube is visible in the subfigure) and additional stresses. The tensile test results, together with the corresponding statistical measures (mean, standard deviation, and coefficient of variation), are summarized in Fig. 9; Table 4. The statistical indicators show generally low scatter and good repeatability for most measured quantities with CoV values typically in the range of 0.01–0.06.

All tested titanium specimens exhibited substantial plastic deformation capacity. However, the *U*_F, max_ for welded butt-joints (mean value is 7.28 mm) is 64% of that measured for the base material (mean value is 11.21 mm). It should be noted that a moderate scatter is observed in *U*_F, max_ for the base material. This can be attributed to the fact that the measured load-displacement curves become nearly horizontal in the vicinity of *F*_max_, which leads to larger variations in the corresponding displacement values. The T-joints showed even smaller ones (mean is 3.29 mm), i.e., about half of the butt-joint values; although, the effective gauge length in the T-joint configuration is half that of the butt-joint specimens (gauge length is approximately 100 mm instead of 200 mm).

**Table 4:** Mechanical properties based on tensile tests.

Specimen		Initial stiffness S_ini_ [kN/mm]	Maximum load F_max_ [kN]	U_F, max_ [mm]	Ultimate strength *R*_m_ [MPa]
Base material (3.7035)	BM-1	33.2	56.58	9.66	645
	BM-2	32.4	59.64	12.93	680
	BM-3	32.6	58.61	11.03	668
	Mean	32.7	58.28	11.21	664
	StD	0.3	1.27	1.34	15
	CoV [–]	0.01	0.02	0.12	0.02
Butt-joint	BJ-1	36.6	39.37	7.40	449
	BJ-2	36.0	39.94	6.90	455
	BJ-3	36.2	39.26	7.54	448
	Mean	36.3	39.52	7.28	451
	STD	0.3	0.30	0.27	3
	CoV [–]	0.01	0.01	0.04	0.01
T-joint	TJ-1	11.7	29.51	3.65	336
	TJ-2	16.8	31.61	2.86	360
	TJ-3	16.9	34.08	3.35	389
	Mean	15.1	31.73	3.29	362
	STD	2.4	1.87	0.33	22
	CoV [–]	0.16	0.06	0.10	0.06

*Note: StD – standard deviation, CoV – coefficient of variation.

Based on the measured initial stiffness values, clear differences can be observed among the investigated joint configurations. The initial stiffness values differ markedly among the titanium specimen configurations. The butt-joints exhibit the highest mean initial stiffness (36.3 kN/mm) due to the weld bead with excess weld metal at the centre of the specimens, followed by the base material specimens (32.7 kN/mm), and the T-joints (15.1 kN/mm). Thus, the base material is approximately 10% less stiff than the butt joints, whereas the T-joints show a reduction of about 54% relative to the butt joints (and about 54% lower than the base material). In terms of repeatability, the initial stiffness values of the base material and butt joint specimens show excellent consistency (CoV = 0.01 for both groups), while the T-joints exhibit higher scatter (CoV = 0.16), primarily due to uncertainties in the top fixturing (using the auxiliary plate with a drilled hole) and the load transfer mechanism (combination of bending and tension).

**Fig. 10:**
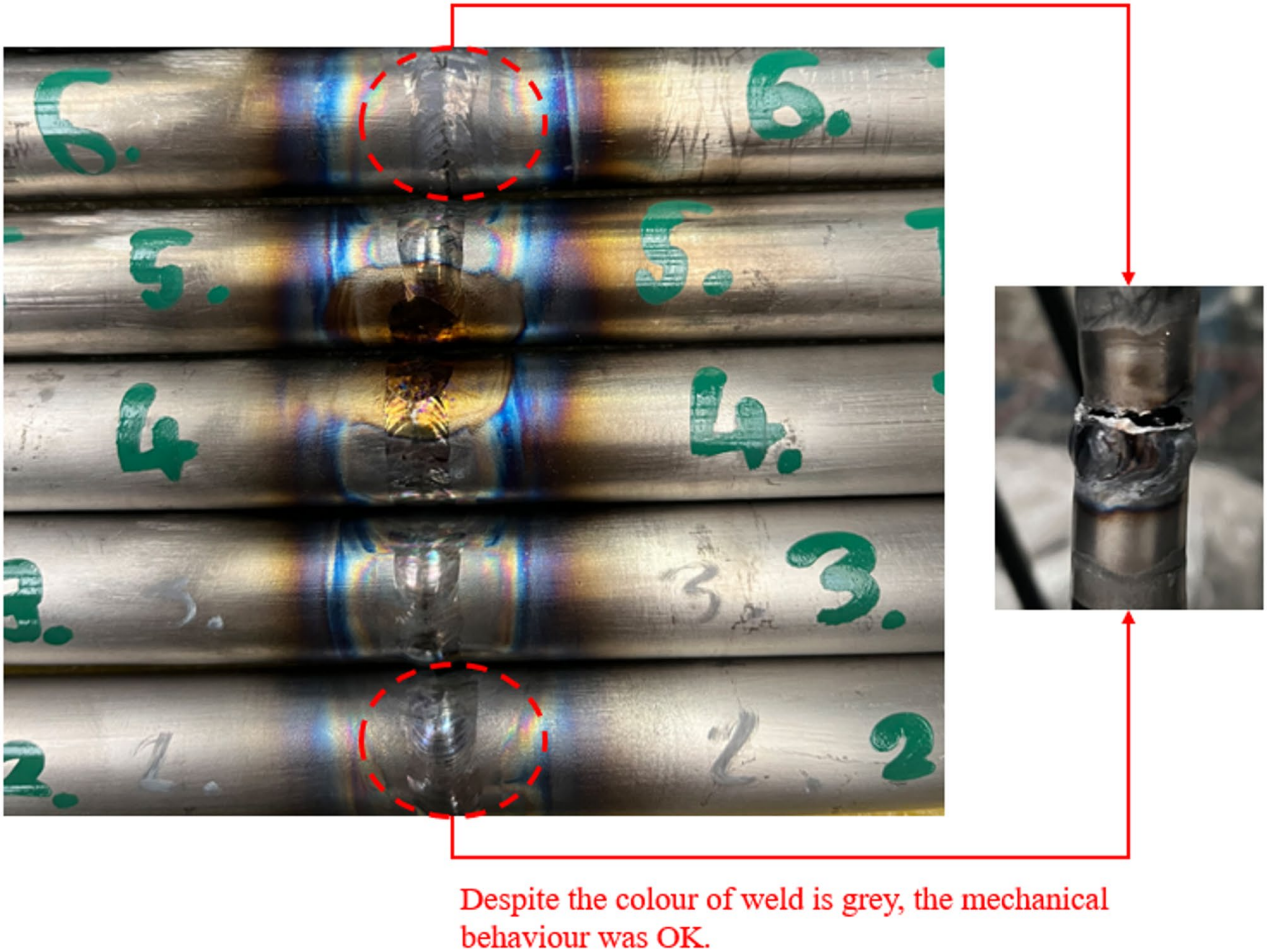
Grey-coloured weld joint and fracture zone after the tensile test.

In addition to the tensile tests, visual inspection of the weld and its vicinity is also carried out. Thus, contaminants, if any, can be observed since it is indicated by colouration. Generally, according to the literature on titanium welding, the basic principle is that the quality of a weld can often be assessed by the colour of the joint and its immediate surroundings. According to^3^, a bright silver weld indicates absence of contaminants, while a golden or straw-like colour indicates that some embrittlement is present. On the other hand, many recommendations or standards, such as AWS D17.1 for aerospace applications, suggest that if the weld appears bright silver, silver, light or dark straw, bronze, or brown, the joint is considered acceptable for weld classes A-C. However, when violet, green, blue, grey or white shades appear, the welded joint should be treated with caution and might be rejected. Nevertheless, the correlation between weld colour, strength, and applicability is not always exact in line with the findings in this study. Weld quality cannot be reliably determined by colour alone. During structural testing, joints with a white-grey appearance show similar properties to those with a bright silver colour. In Fig. 10, the fracture zone of a grey-coloured weld joint is presented, even though the joint demonstrated adequate tensile performance. The Authors believe that future research should more thoroughly investigate the relationship between weld colour, load-bearing capacity, and overall joint quality.

### Peel tests and combined peel-tensile tests

**Fig. 11:**
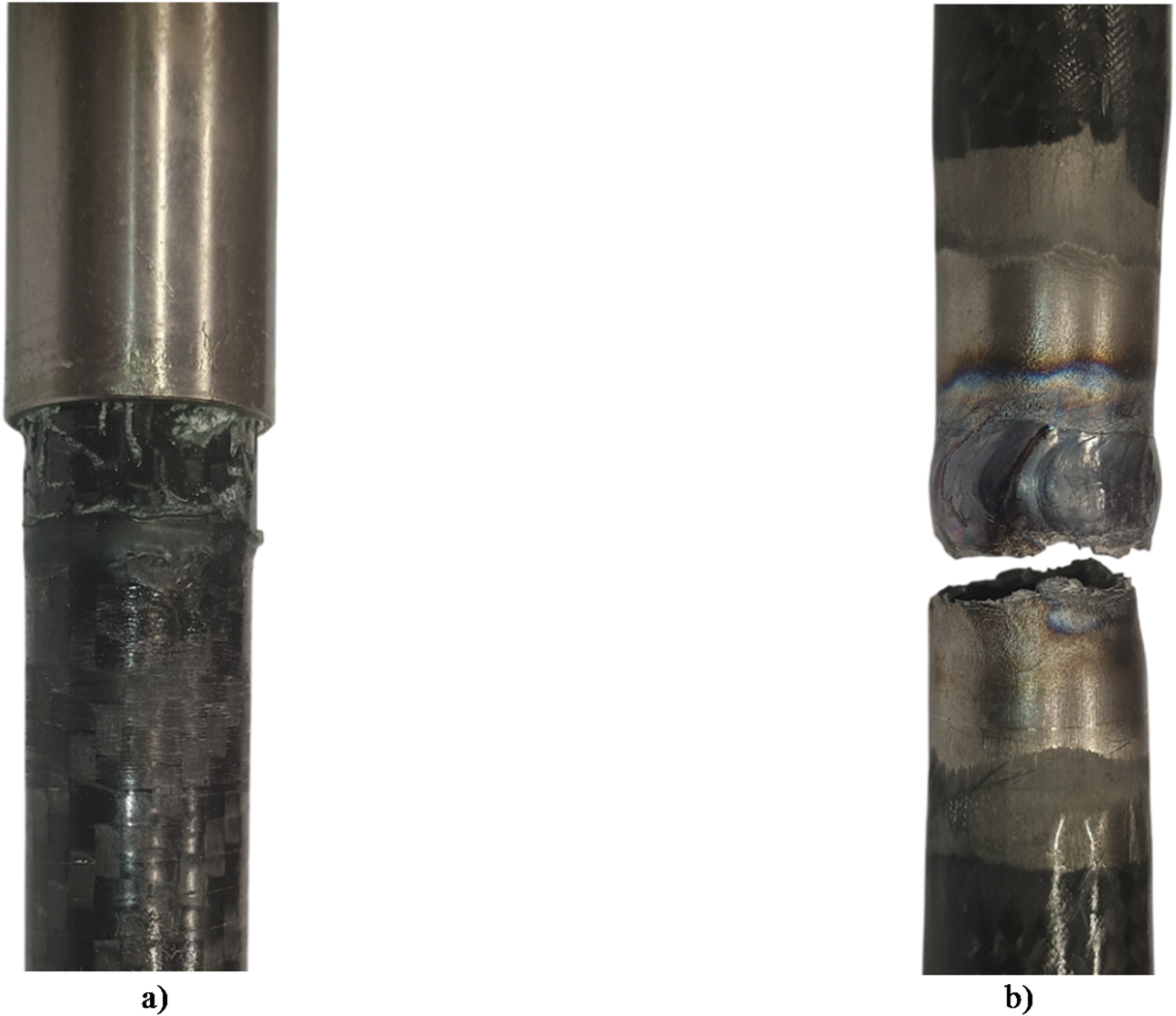
Typical failure modes of (**a**) bonded joint and (**b**) hybrid joint.

**Fig. 12:**
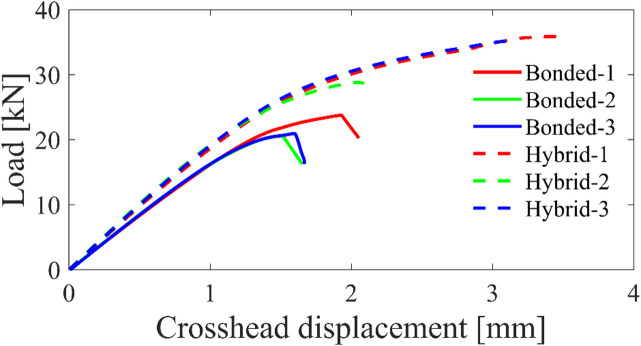
Measured load-displacement curves for peel tests and combined peel-tensile tests.

Peel tests of the three bonded specimens (Bonded-1 to Bonded-3) resulted in brittle interface failure of the adhesive bond (Fig. 11a) with a massive reduction, approximately 45%, in load-bearing capacity compared to their welded counterparts (BJ-1 to BJ-3) as the mean maximum load reduces to 21.75 kN from 39.52 kN. Load-displacement curves are plotted in Fig. 12. The measured peel strengths exceed the value specified in the technical datasheet, with a minimum of 12.99 MPa compared to the specified 11 MPa. The coefficient of variation of derived peel strengths is only 0.07, indicating a highly uniform bonding process and consistent adhesive quality. This configuration exhibits reduced ductility: the *U*_F, max_ has a mean value of 1.67 mm over a gauge length of 180 mm, with a coefficient of variation of 0.11, and failure occurs abruptly after reaching *F*_max_.

A total of three hybrid specimens is tested (denoted as Hybrid-1 to Hybrid-3) during combined peel-tensile testing. The bonded joint is not the weakest link in the hybrid joint; the failure occurs in the heat-affected zone near the weld (Fig. 11b). Failure occurs at a lower displacement level due to the reduced deformation capacity of the shorter ductile metallic part (only free length of 60 mm at the centre) compared to welded butt-joints (BJ-1 to BJ-3), combined with the brittle behaviour of the CFRP tubes. The measured load-displacement curves are plotted in Fig. 12 together with the peel tests. The results indicate that the hybrid bonded-welded joints show approximately an average reduction of 16% in load-bearing capacity compared to the butt-welded tubular sections: the mean maximum load reduces to 33.29 kN from 39.52 kN. However, it is underscored that the minimum *F*_max_ for these joints is comparable with the minimum guaranteed value by the manufacturer for the base material (*F*_min_ = *R*_m, min_ · *A*_Ti, tube_ = 87.7 mm^2^ · 390 MPa = 34.21 kN). The mean *U*_F, max_ is 2.84 mm (noting that this value includes/smears local deformations near the failure region) over a gauge length of 320 mm. This indicates ductility comparable to that of the bonded specimens, as the normalized displacements are essentially identical (1.67/180 = 0.009 and 2.84/320 = 0.009). The hybrid bonded-welded joints show intermediate stiffness values, with a mean of 19.9 kN/mm, while bonded joints show a moderately reduced stiffness, averaging 17.2 kN/mm. This corresponds to a stiffness reduction of 45% and 53%, respectively, relative to the butt-welded joints.

**Table 5:** Mechanical properties based on peel tests and combined peel-tensile tests.

Specimen		Initial stiffness S_ini_ [kN/mm]	Maximum load F_max_ [kN]	U_F, max_ [mm]	Peel strength τ_peel_ [MPa]	Ultimate strength *R*_m_ [MPa]
Bonded joint	Bonded-1	16.9	23.75	1.92	15.00	–
	Bonded-2	17.3	20.56	1.51	12.99	–
	Bonded-3	17.3	20.95	1.59	13.23	–
	Mean	17.2	21.75	1.67	13.74	–
	STD	0.2	1.42	0.18	0.90	–
	CoV [–]	0.01	0.07	0.11	0.07	–
Hybrid joint	Hybrid-1	19.4	35.87	3.35	–	409
	Hybrid-2	20.4	28.80	2.05	–	328
	Hybrid-3	20.0	35.19	3.11	–	401
	Mean	19.9	33.29	2.84	–	379
	STD	0.4	3.18	0.56	–	36
	CoV [–]	0.02	0.10	0.20	–	0.10

*Note: StD – standard deviation, CoV – coefficient of variation.

The derived CoVs indicate low scatter and good repeatability (< 0.10) for all the analysed characteristics in general (Table 5). Most importantly, excellent repeatability and consistency are shown for the initial stiffness of the specimens with CoV = 0.01 and 0.02 for bonded and hybrid specimens, respectively. However, *U*_F, max_ shows moderate scatter with CoV = 0.20 for hybrid joints. It is noteworthy that Hybrid-2 exhibits outlier behaviour in terms of ductility (which is not of utmost importance due to the application of CFRP) and maximum load-bearing capacity.

### **Three-point bending tes**ts

**Fig. 13:**
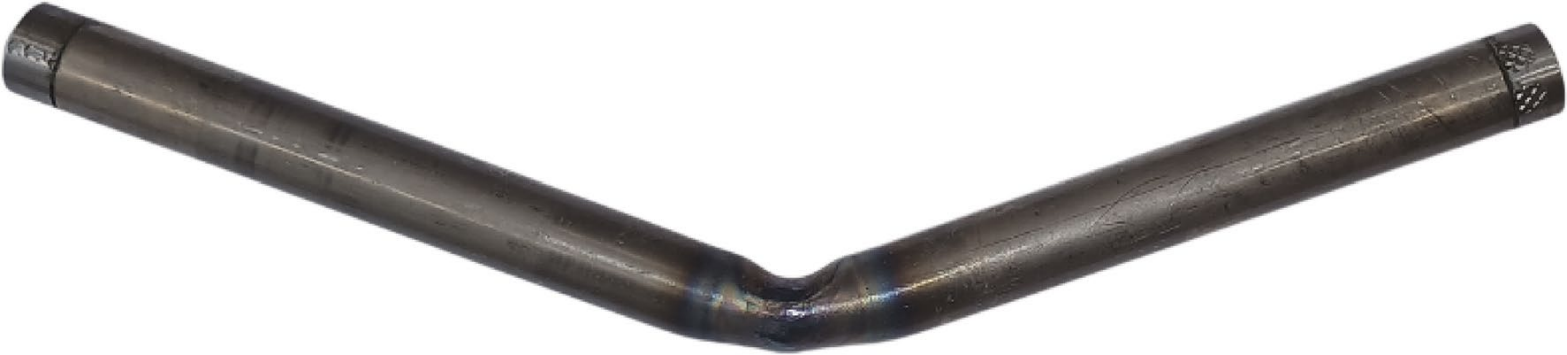
Deformed shape of a butt-welded joint subject to three-point bending test.

**Fig. 14:**
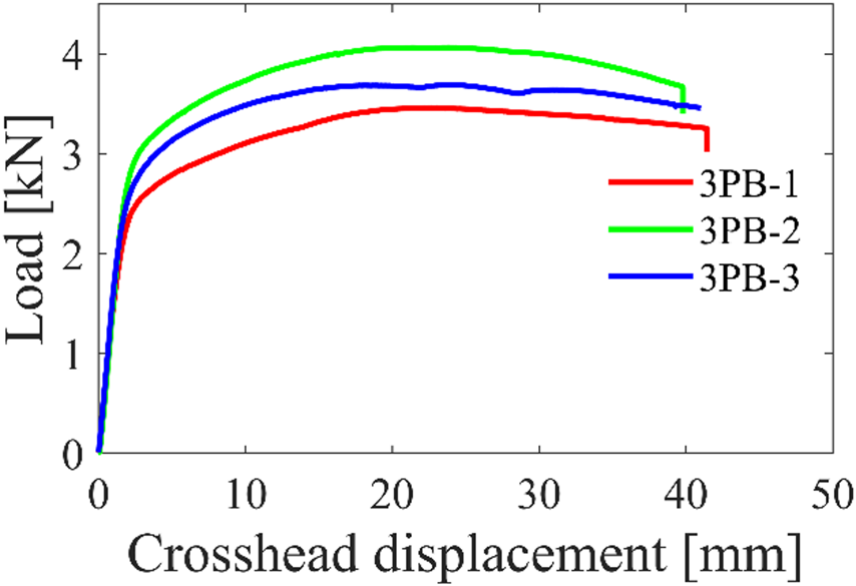
Set-up for three-point bending tests, and corresponding measured load-displacement curves.

The three-point bending tests confirm the adequacy of the applied welding process of the butt-welded tubular sections, as no fractures or cracks are observed (Fig. 13). Only the welded joints are subjected to three-point bending tests, as the structural nodes of the lightweight UAV spatial truss are exclusively welded, and therefore the bending-type stresses associated with load transfer and eccentricity arise primarily at these locations. Figure 14 presents the load-displacement curves, which clearly demonstrate significant plastic deformation capacity. Mean of maximum load *F*_max_ is 3.74 kN based on the three tested specimens (3 PB-1 to 3 PB-3), while CoV is 0.03–0.07 for the analysed characteristics meaning good consistency. However, it is highlighted as well that the derived initial stiffness values here are not comparable with the ones in Tables 4 and 5 for the specimens subject to tensile loading due to different loading mechanism. It should be noted that the three-point bending test conducted in this study does not fully comply with the procedure specified in EN ISO 8491, as the angle of bending applied here deviates from that prescribed in the standard. Nevertheless, the test provides reliable determination of load-bearing capacities. Test results, together with the statistical data, are summarized in Table 6.

**Table 6:** Mechanical properties based on three-point bending tests.

Specimen	Initial stiffness S_ini_ [kN/mm]	Maximum load F_max_ [kN]	U_F, max_ [mm]
3 PB-1	1.53	3.46	22.52
3 PB -2	1.61	4.06	21.40
3 PB -3	1.63	3.69	24.15
Mean	1.59	3.74	22.69
STD	0.04	0.25	1.13
CoV [–]	0.03	0.07	0.05

### Results of macroscopic and microscopic examinations

**Fig. 15:**
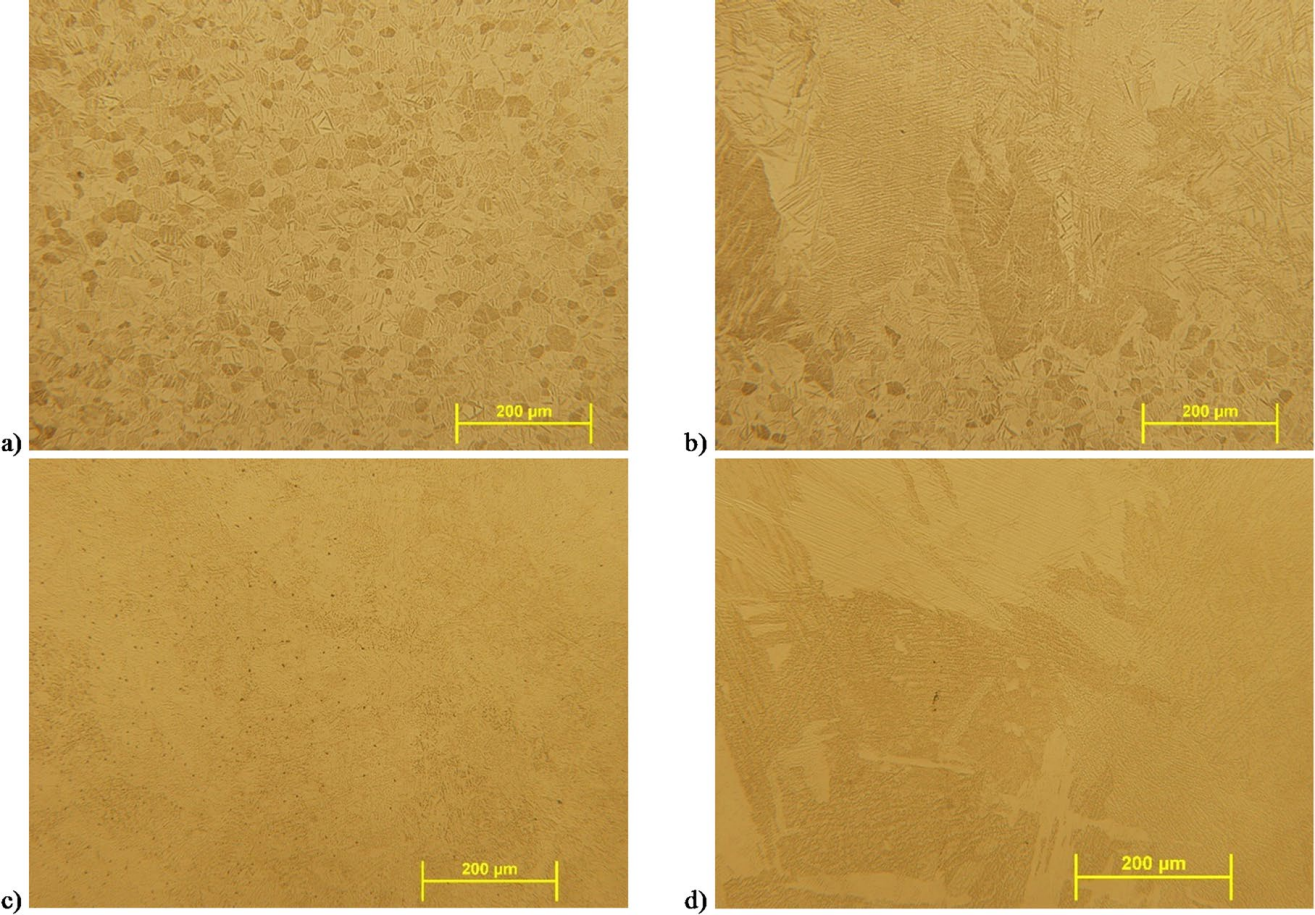
Optical micrographs for (**a**) base material, (**b**) boundary of BM and HAZ, (**c**) HAZ, and (**d**) weld metal.

Optical micrographs are presented in Fig. 15. The base material exhibits a fine and homogeneous equiaxed α-phase microstructure with hexagonal close-packed (HCP) crystal structure (also with annealing twins observed locally within some grains), characteristic of commercially pure titanium (Fig. 15a). The α-phase is stable up to the β-transus temperature, where it transforms into a body-centred cubic (BCC) structure, i.e., the β-phase. The α-β transition temperature is affected by the alloying elements such as iron or chromium and can reduce the β-transus temperature. At the boundary of BM and HAZ (Fig. 15b), the fine equiaxed base material microstructure gradually transitions into a coarsened region with emerging lamellar/acicular features, also indicating the onset of a Widmanstätten-type (‘basket-weave’) structure. The HAZ (Fig. 15c) exhibits a thermally altered microstructure characterized by a coarsened transformed α structure with a ‘basket-weave’ morphology. The original equiaxed morphology entirely disappears, indicating substantial microstructural modification due to the welding thermal cycle. In the weld metal, a distinctly coarse lamellar structure is observed, consisting of colonies of larger acicular α laths with varying orientation formed during cooling from the β phase field. Significant grain coarsening in both the HAZ and WM (Fig. 15d) is in line with^3^ for fusion welded joints. This microstructural evolution supports the assumption on softening behaviour discussed in Sect.  3.1, as evidenced by the tensile test outcomes and the corresponding failure locations next to the weld beads.

**Fig. 16:**
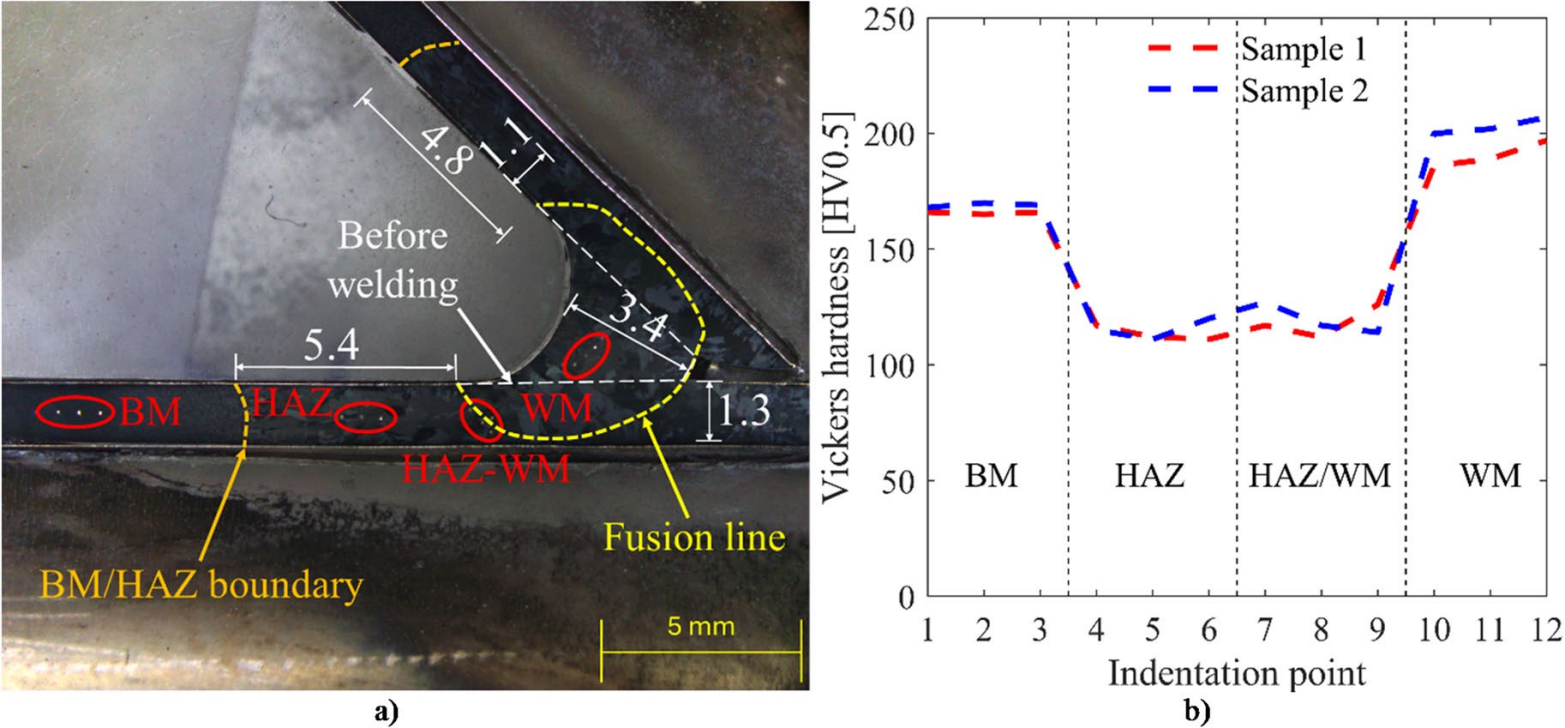
(**a**) Joint morphology with location of indentations, (**b**) Vickers hardness values for the analysed samples.

Stereomicroscopic observations are used to characterize the macroscopic weld morphology of the tubular Y-joint, including weld bead geometry, penetration depth, fusion line location, and the presence of visible defects. A macrograph is presented in Fig. 16a, together with the locations of the hardness indentations. The concave crown side appears smooth, with no visible undercuts or cracks; however, incomplete penetration is observed on the root side. The weld metal area is 18.3 mm^2^, with fusion penetration depths of 1.1 mm and 1.3 mm, and an actual throat thickness of 3.4 mm. The figure indicates that the heat-affected zone is relatively extensive.

Hardness is measured on the inner side of the welds for both samples. The Vickers hardness values are illustrated in Fig. 16b. Indentation points #1–3 denote the base material, #4–6 are in the heat-affected zone, while #7–9 are at the boundary of HAZ and WM and #10–12 are in the weld metal. The heat input inevitably causes a significant change in grain size, which in turn results in variations in hardness. The Vickers hardness measurements reveal a clear variation across the different regions of the welded joint. In the base material, the hardness values range between 165 and 170 HV0.5, showing very consistent behaviour for both analysed samples (reference hardness is 175 HV according to the inspection certificate). Moving towards the HAZ, a pronounced reduction in hardness is observed, with values decreasing to approximately 111–120 HV0.5, indicating local softening in this region. In the HAZ/WM transition, the hardness is basically identical with the HAZ, falling within the range of 112–127 HV0.5. In contrast, the weld metal exhibits the highest hardness values, ranging from 186 to 207 HV0.5, which is significantly higher than both the base material and the adjacent heat-affected zone.

The HAZ exhibits reduced hardness compared to the base material, in agreement with the Hall-Petch relationship, as significant grain growth lowers strength and hardness. Reduced hardness can be explained with the relatively low thermal conductivity of titanium, approximately λ = 7 W/(m∙K)^64^, thus the heated area cools more slowly. Below the β-transus temperature, time- and temperature-dependent diffusion processes are slower: slow cooling results in a coarse lamellar structure and softening in the HAZ as described previously in^3^.

Titanium alloys are generally welded with matching filler materials; however, for commercially pure titanium (Grades 1–4), a filler wire of one lower strength grade may be selected^3^. Accordingly, an intentionally undermatching filler material is used in the present study. Nevertheless, tensile tests show that the ultimate strength of the base material is substantially higher than the minimum guaranteed strength of the filler metal. In contrast, the present results show that the Vickers hardness, and correspondingly the ultimate strength, of the weld metal is higher than that of the base material. It indicates slight hardening in the weld metal, which is attributed to possible atmospheric gas pick-up, even welding is performed in a purge chamber under argon shielding. It is in line with the findings about hardening in the weld metal as noted in^3^.

## Structural integration and scalability

From an engineering perspective, strength reduction of the hybrid bonded-welded joints relative to the butt-welded joints represents a favourable compromise. Although their initial stiffness is lower than that of the fully titanium butt-welded joints, the hybrid configuration is well suited for transition zones between titanium and CFRP members in lightweight UAV or aerospace frames. Accordingly, fully welded titanium joints remain preferable for primary highly loaded metallic nodes and truss members, whereas hybrid joints are more suitable for lower-force connections, offering a practical balance between weight efficiency, manufacturability, and structural reliability.

At the level of tubular space truss of the UAV, the joint behaviour must be considered together with force redistribution, stiffness differences, and multi-axial loading. While the present tests characterize the local response of the individual joint types, their performance in a redundant frame may be affected by secondary bending, geometric imperfections, and assembly tolerances. In this context, the lower stiffness of hybrid and bonded joints may increase global compliance, whereas welded titanium joints provide greater local rigidity. The fact that the hybrid joints fail in the heat-affected zone rather than at the adhesive interface suggests that, with proper detailing, they can serve as reliable metal-to-composite transition joints. Further validation at larger structural scale is needed including full space truss testing and numerical modelling to assess joint interaction, progressive failure, and sensitivity to manufacturing tolerances.

## Conclusions

A comprehensive experimental research program is carried out focusing on the load-bearing capacity and mechanical behaviour of welded, bonded and hybrid welded-bonded joints, subject to quasi-static loading at room temperature, made of thin-walled 3.7035 titanium alloy (Grade 2) and CFRP tubes. The experimental results reveal the following key findings:

- The titanium base material exhibits ultimate strengths of 645–680 MPa, significantly above the minimum guaranteed value of 390 MPa.
- Welded titanium butt-joints show a ~ 30% reduction in strength compared to the base material; however, still exceeding the DIN 17,861 requirement, with ductile failures occurring in the heat-affected zone.
- Welded T-joints reach lower strength values (~ 20% below butt-joints), mainly due to the non-pure tensile loading conditions.
- During tensile testing, welded joints with a white-grey appearance show similar properties to those with a bright silver colour.
- Bonded joints exhibit about 45% lower load-bearing capacity than welded joints, due to brittle interface failure of the adhesive bond, while hybrid welded-bonded joints show only ~ 16% reduction and maintain strengths comparable to the guaranteed minimum for the base material.
- Three-point bending tests confirm the adequacy of the welding process, as no cracks or fractures are observed.
- Hybrid and bonded joints show 45% and 53% reduction in mean initial stiffness compared to butt-welded joints.
- Microstructural examinations and hardness measurements reveal softening in the heat-affected zone due to grain coarsening, which explains the observed failure locations during testing.

Although beyond the scope of the present study, the authors intend to focus on the behaviour of bonded and hybrid welded-bonded joints under dynamic and cyclic loading in future work, while also incorporating numerical modelling to describe the load transfer mechanisms. Further investigations under extreme environmental conditions (e.g., low temperatures) are also considered a potential direction for further research.

## Data Availability

Data will be available on request.
